# Child Malnutrition in Pakistan: Evidence from Literature

**DOI:** 10.3390/children5050060

**Published:** 2018-05-04

**Authors:** Muhammad Asim, Yasir Nawaz

**Affiliations:** 1 Population Research Center, University of Texas, Austin, TX 78712, USA; 2 Department of Sociology, University of Sargodha, Sargodha, Punjab 40100, Pakistan; yasir.manj@uos.edu.pk

**Keywords:** child malnutrition, undernutrition, nutritional status of children, developing country, Pakistan

## Abstract

Pakistan has one of the highest prevalences of child malnutrition as compared to other developing countries. This narrative review was accomplished to examine the published empirical literature on children’s nutritional status in Pakistan. The objectives of this review were to know about the methodological approaches used in previous studies, to assess the overall situation of childhood malnutrition, and to identify the areas that have not yet been studied. This study was carried out to collect and synthesize the relevant data from previously published papers through different scholarly database search engines. The most relevant and current published papers between 2000–2016 were included in this study. The research papers that contain the data related to child malnutrition in Pakistan were assessed. A total of 28 articles was reviewed and almost similar methodologies were used in all of them. Most of the researchers conducted the cross sectional quantitative and descriptive studies, through structured interviews for identifying the causes of child malnutrition. Only one study used the mix method technique for acquiring data from the respondents. For the assessment of malnutrition among children, out of 28 papers, 20 used the World Health Organization (WHO) weight for age, age for height, and height for weight Z-score method. Early marriages, large family size, high fertility rates with a lack of birth spacing, low income, the lack of breast feeding, and exclusive breastfeeding were found to be the themes that repeatedly emerged in the reviewed literature. There is a dire need of qualitative and mixed method researches to understand and have an insight into the underlying factors of child malnutrition in Pakistan.

## 1. Introduction

There are 165 million malnourished children under five years around the globe [1]. Malnutrition accounts for at least half of all childhood deaths worldwide [2,3]. Child malnutrition is generally only a problem of developing and underdeveloped countries [4]. Malnutrition is the fundamental cause of morbidity and mortality among the children [5]. Almost half of the mortality in children around the globe is attributed to undernutrition [6]. It also poses a risk to children’s physical and mental development, which results in poor academic achievement [7]. Adequate nutrition is indispensable to ensure a strong immune system and proper physical and intellectual development in early childhood [8,9].

It has been estimated that 170 million (30%) of children under the age of five in the world are moderately or severely stunted, and 110 million (19%) are moderately or severely underweight [10]. Almost half of all stunned children reside in Asia, 51 million (8%) children under five years of age are wasted, and two thirds of all wasted children live in Asia [11]. Malnutrition affects the future health and socioeconomic development of children and the dynamic prospective of the society. Pakistan has been reported to have one of the highest levels of prevalence of child malnutrition compared to other developing counties [12]. According to the National Nutrition Survey, 33%of all children were underweight, nearly 44% were stunted, 15% are wasted, 50%were anemic, and 33%were anemic (iron deficiency). In the last two decades, there has been a little reduction in the prevalence of child malnutrition in Pakistan compared to other developing countries [13].

Despite economic and social development, childhood malnutrition still remains a major public health and social problem in less developed countries [3,14,15]. The contributing factors in childhood malnutrition are low birth weight, inadequate breast feeding and exclusive breastfeeding, inappropriate complementary feeding, maternal education, lack of proper knowledge of nutrition, micronutrient intake, parity, birth spacing, household socioeconomic status, food insecurity, poor sanitation, vaccination, and infectious diseases [16,17,18,19,20]. Pakistan is among the countries in the world with the highest rates of child malnutrition, and its progress in child nutrition and health remains slower than in other South Asian countries [21,22,23].

## 2. Aim and Objectives of the Review

Malnutrition is the underlying cause of child morbidity and mortality in Pakistan. It has not been the priority issue of government of Pakistan to overcome the malnutrition in children. The aim of this narrative review is to collate and synthesize the published data to understand the problem as a whole. The dearth of studies on child malnutrition in Pakistan has evoked the researcher to search for and review the existing relevant literature. The specific objectives of this review article were:

(i) to identify about the range of methodologies and methods to access the children malnutrition;
(ii) to highlight the emerging themes to address the children malnutrition;
(iii) to identify the geographical areas that are neglected in this research in Pakistan; and
(iv) to provide recommendations for future work.

## 3. Methodology

Malnutrition is widely known as under-nutrition until and unless it is not specified [24]. Malnutrition in children is assessed through stunting, wasting, and underweight in children less than five years of age [25]. Stunting is chronic malnutrition, underweight is acute malnutrition, and wasting is the combination of acute and chronic malnutrition. Pakistan has been divided into four administrative provinces: Punjab, Sindh, Khyber Pakhtunkhwa (KPK), and Baluchistan. More than two-thirds of the population of Pakistan live in rural areas, where the prevalence of poverty is more common. Most of the rural people are engaged in agriculture and agricultural labor, taking care of livestock, and informal business and have the highest prevalence of malnutrition among their children.

As the topic is public health and anthropological relevance, various databases were utilized, including PubMed, Medline, NCBI, CINAHL, Scopus, and social science citation index. A number of keywords and combination of keywords were used to locate the relevant literature, such as “child malnutrition in Pakistan”, “child undernutrition in Pakistan”, “nutritional status of children in Pakistan”, “child health and nutritional status in Pakistan”, and “child health situation in Pakistan”. Furthermore, the list of references of relevant papers was also reviewed to access for more relevant literature on child malnutrition. 

### Inclusion and Exclusion Criteria

**Figure 1:**
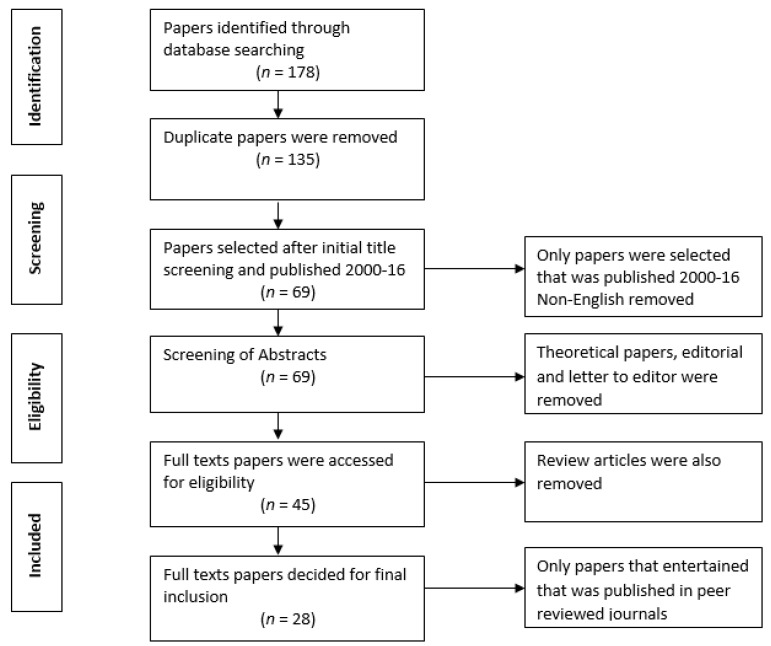
The process of selection of relevant papers is given criteria.

The most relevant and current published papers in between 2000–2016 were included in this methodological review article. The research papers that contain the data related to Pakistan were assessed. The review papers, case studies, and letters to the editors were not entertained in this study. At the first stage, through different keywords, 178 papers were searched through different search engines using medical subject heading. At the second stage, titles and abstracts were screened for inclusion in the current study, and duplicate and irrelevant papers (review papers, opinion, and letter to editor) were discarded. At the third stage, 69 papers were selected to review the title of the papers. At the fourth stage, the whole abstracts of the selected papers were reviewed and screened, and at the fifth stage, the decision was made to either include or exclude to review the whole article on the basis of full text papers. At the final stage, 28 papers were considered relevant and included for accomplishing this study. Narrative review is a method used to describe trends in the research field by calculating how many studies have used certain research methodologies, where they were carried out and what are the associated factors with problem under investigation. In a narrative review, the statistical analysis is not possible because the previously published studies were diverse in their objectives and questions. Narrative reviews do connect information into themes. The meta-analysis is only suitable for statistical analysis. Meta-analysis can only be undertaken when studies address the same question and administer the intervention in a similar manner or measure the same outcomes [26]. There was no any disagreement between the authors in the whole procedure of inclusion and exclusion of the papers in this study. The systematic method of selecting the relevant papers is presented in Figure 1.

## 4. Results

### 4.1. Methodological Findings

It was observed that in four papers (Table 1), body mass index (BMI) was used to access the malnutrition status of children. Two authors used weight and height through Jelliffe’s classification, one paper used Harvard standard, and two papers used the rapid assessment approach mid upper arm circumference (MUAC) for assessing the malnutrition among children. 

In 12 papers, the target population of the children was school-age (more than five years), and 15 papers focused on less than five years’ children in the assessment of malnutrition. Different national and international reports have warned that first 1000 days are the window of the opportunity to strengthen the nutritional status of the children [1,26,27,28,29]. Only two papers focused on the most vulnerable population of less than two years for the assessment of the malnutrition. Out of 28 studies, 13 studies were community-based, nine were school-based, four were hospital-based, and one was from secondary data analysis. Mostly, the studies were community-based and school-based for the assessment of the child nutrition in Pakistan. Out of 28 papers, 10 papers were published in international journals, and the rest of them were published in different Pakistani journals. 

Different other cross-sectional research and regional studies presented varied results in term of stunting, wasting, and underweight according to their methodology and geographical area. The prevalence of child malnutrition according to the studies under reviewed is discussed in Table 2. In KPK, district Nowshera, Ali [30] found that 12.5% children were stunted, 15% were underweight, and 7% were wasted. Another study from KPK, Afridi accounted that 14% children are underweight, 8% were underweight and stunned in Swat [31]. In a study from Quetta, Baluchistan [32], it was reported that one out of two (48%) children were stunned and 10% were wasted. In another study Ansari [33] highlighted that 22% children were stunned, 10% were wasted, and 24% were underweight in urban areas of Pakistan. Similar finding was also presented by the Batool [34] that the prevalence of stunting was 46% and underweight was 25% and 18% was under weight. In Sindh province, the situation of children in term of stunting, wasting, and underweight is alarming according to different studies. Nisar reported that 61% of children were stunned, 54% were underweight, and 47% were wasted in rural Sindh [35]. Similar findings were observed by Shah, who found that 26% children were wasted, 55% were stunned, and 15% were both wasted and stunned in hospitalized children [36].

### 4.2. Coverage of Geographical Area

**Figure 2:**
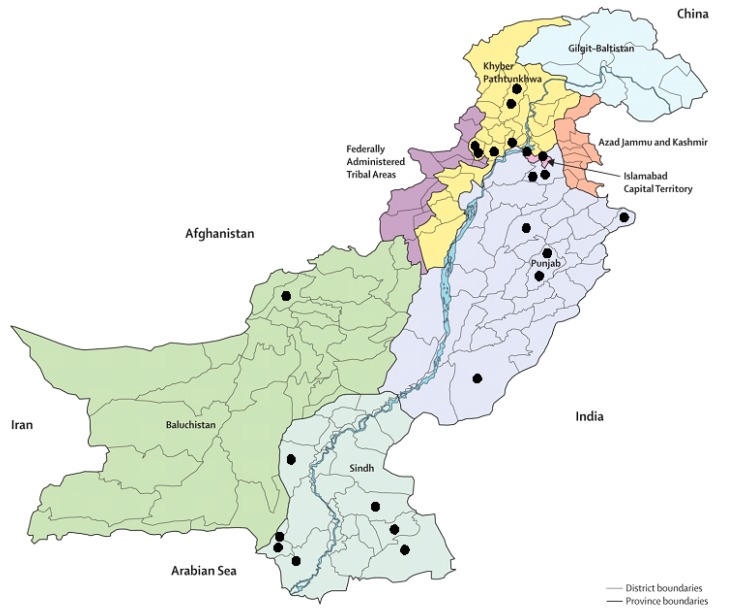
Coverage of geographical areas in the studies reviewed. The total number does not equal the total number of studies as certain studies focused on more than one area.

Figure 2 shows a map of Pakistan and geographic areas under review in this study. Most of the studies on child malnutrition focused on Sind and Punjab provinces. In Sind province, most of the studies focused in the rural areas. In the Punjab, most of the studies were conducted in central Punjab, and only one study covered the area of southern Punjab. Punjab has more than half of the population of Pakistan but still has many districts and divisions that have not yet to be studied, to know about the severity of the problem. In KPK, six researches were found on child malnutrition, two from Peshawar, and two from Swat. Two studies were conducted in the capital city (Islamabad). However, only one study has been carried out in the provincial capital Quetta, Baluchistan, and none of the research on child malnutrition was carried out in Gilgit Baltistan, Azad Jammu Kashmir, and Federally Administrated Tribal Areas (FATA).

### 4.3. Child Age

Different studies have highlighted that the severity of malnutrition occurs in a particular age group of children. Laghari [37] and Gul and Kibria [38] pointed out that the prevalence of malnutrition is considerably higher in children less than two years of age. Studies from Sindh, Tharparkar, and Umerkot also exhibited similar finding that the highest prevalence of malnutrition was found in children less than two years of age [39]. Nisar assessed the nutritional status of hospitalized children and found that children less than two years of age had a higher prevalence of nutritional anemia and poor nutritional status in the capital city of Pakistan [35]. Nuruddin [40] also pointed out that the prevalence of wasting, stunting, and underweight were higher in the age group of those less than two years of age. 

### 4.4. Rural Urban Disparities 

There is rural and urban disparity in the prevalence of child malnutrition across the world. Mushtaq pointed out that in rural areas the prevalence of child malnutrition was higher compared to urban areas [41]. The burden of malnutrition was lower in urban areas as compared to rural areas in Pakistan. Similar finding was presented by Anwer, who stated that children belong to rural areas are highly vulnerable of all types of malnutrition. In rural areas of Faisalabad, 65% of children were underweight, 41% were stunned, and 33% were wasted [42]. According to the national nutrition survey of Pakistan, acute and chronic child malnutrition were significantly higher in rural areas as compared to urban areas across the country [13].

### 4.5. Gender

Gender is an important indicator of child malnutrition. Khattak found there was no significant difference in stunting and wasting in male and female children [43]. Many other studies, including Khan [44], Mushtaq [41], and Shah [36], also ascertained that stunting and thinness was not associated with gender. Laghari [37] stated that severe malnutrition was reported higher in females than male children. Similar findings were also presented by Ansari [33], who stated that female children were three times more likely to be stunned than male children. Khuwaja [45] and Batool [34] also pointed out that female children were found more likely to be stunned as compared to male children. Another perspective was also presented in some research where the highest prevalence of malnutrition was found in boys as compared to girls. Fryarand and Ogden determined that boys were 1.5 times more likely to be underweight than girls [46]. There was a greater likelihood ratio of undernutrition in boys as compared to girls [47]. According to the Pakistan Demographic and Health Survey [48], the prevalence of stunting was slightly higher (51%) in boys than in girls (45%).

## 5. Discussion

### 5.1. Methodological Issues

Table 1 depicted that similar methodology had been used to collect data on child malnutrition in previously published papers in Pakistan. However, most of the researchers have conducted the cross sectional quantitative and descriptive studies, through structured interviews for assessment of child malnutrition. Kroger pointed out that this is the best technique to overcome the interviewer’s influences [60]. Various medical and social indicators are being used to examine the nutritional status of children i.e., MUAC, anthropometry, calories intake method, hemoglobin test, and through skin fold thickness [61]. It was found that most of the research used anthropometric assessments include wasting (weight-for-height), stunting (height-forage), and underweight (weight-for-age) by the National Center for Health Sciences (NCHS)/World Health Organization (WHO) for assessment of child nutritional status [62,63]. The WHO classification is widely used as the benchmark for assessing the malnutrition children under the age of five across the globe. WHO classification is currently the most accepted method for assessing malnutrition in children [64]. This criterion was formulated and updated, keeping in view the growth standard of the six courtiers across the world i.e., Ghana, Brazil, India, Oman, the United States of America (USA), and Norway through a multicenter growth reference study in 2006 [65]. WHO classification for child malnutrition provides comprehensive study of all types of child malnutrition. This is why most of the researchers used WHO classification to assess the child malnutrition in their studies in Pakistan. However, the MUAC technique is used as a quick and easy method to identify the malnutrition in under five years children [66,67,68]. MUAC is a better indicator to observe the nutritional status of children in emergency situations [69]. Some other anthropometric indicators have been used for the assessment of child’s nutritional status are Jelliffe’s classification, Gomez’s classification, and weight for age using Harvard standard. Another social indicator dietary diversity or calories intake method and hemoglobin level biomedical method are also used for the nutritional assessment of children. However, in different papers, BMI was also used for nutritional assessment especially for children of more than five years of age. Dietary diversity or calories intake method is a complex method to access the malnutrition status among children [70]. Therefore, the researchers could not find any study for the assessment of nutritional status among children through calories in take method in previously published papers in Pakistan from 2000–2016. The nutritional status of infants and children has proved almost impossible to estimate accurately through the calorie intake approach. For these age groups, it is generally agreed that anthropometric method provides more reliable estimates [71]. Therefore, most of the researchers utilized the anthropometric methods to access the nutritional status of children. 

### 5.2. Prevalence of Malnutrition among Children

According to the latest national survey, 31.5% children were stunned, 45% were underweight, and 10.5% were wasted [47]. Similar results were presented in another national study that 15% children were wasted, 43% were stunting, and 31% were underweight [13]. According to United Nations International Children’s Fund (UNICEF), the global stunting rate is 23%, underweight is 16%, and wasting is 7%. Keeping in view the global prevalence of undernutrition, Pakistan has a higher burden of all the types of undernutrition in all the previously published research reviewed in this study. The government should launch policy measures to reduce the burden of all types of malnutrition in Pakistan. The prevalence of all types of child malnutrition is varied in different research under review in this study due to different sample size, geographical areas, type of studies, and different measuring techniques of child malnutrition. The prevalence of child malnutrition according to the studies under review is presented in Table 2. 

### 5.3. Associated Factors with Malnutrition

Most of the researchers highlighted that socioeconomic, demographic, and lifestyle factors are responsible of malnutrition in children. Associated factors with child malnutrition according to the studies under review are briefly explained in Table 2. According to Khattak et al. (2010) [43], there is a strong association of malnourished children with family size, household income and number of children in a family. Gul and Kibria [38] stated that socioeconomic factors leading towards malnutrition were mother’s illiteracy, younger mothers, and multiple parities. Batool [34] and Mushtaq [55] pointed out that poverty, lower education, low income, and overcrowded houses were the associated factors of children malnutrition. Malnutrition is directly associated with large family size, mother’s illiteracy, and poverty. In Pakistan, the typical family size is almost seven members per household, indicating one member is fulfilling the social, economic, and biological needs of the whole family. Almost 40% of the Pakistani population lives under the poverty line. Ali pointed out that illiteracy, large family size, lack of or exclusive breastfeeding, early weaning, and poverty were the associated factors for child under nutrition [30]. Mahmood [49] pointed out that malnutrition is significantly associated with maternal literacy and the presence of family members with special needs. Households with uneducated parents tend to have low income and have large families where household head not able to spend more on food, which leads to growth failure in children. The uneducated parents also have not enough awareness and knowledge about the importance of child exclusive breastfeeding and initiation of complementary feedings that are prerequisites for child health and nutrition.

Ansari [33] pointed out that, in those houses where knowledge of childcare practices are insufficient, food insecurity exists, lack of mother’s education, and large family size were the major determinants of child stunting. Ullah [53] found that teenage pregnancy, large family size, and lack of child vaccination were major associated factors of child malnutrition in Swat. Shah [36] pointed out that stunned children were more likely to live in a house with more than three people sharing one room, mud houses, have incomplete child vaccinations, less income, and no formal education in the rural areas of Pakistan. Hasnain et al. (2010) [56] found that a birth interval of less than two years was the leading factor of child stunting in Dadu Sindh. The prevalence of stunting and wasting among children was more common in a nuclear family [52]. 

### 5.4. Child Age

Different national and international reports have warned that the first 1000 days are the window of the opportunity to strengthen the nutritional status of children [58,59,60]. Different published studies have clearly depicted that the higher burden of child malnutrition can be traced in children less than two years of age. The first phase of malnutrition starts in the gestational period due to lack of proper diet of women during pregnancy. Low birth-weight babies have higher risk of morbidity, mortality, and being malnourished. The second phase of malnutrition starts in infancy or the growth faltering stage, where children remain highly dependent on breastfeeding and complementary feeding. Therefore, it is suggested that more focus should be given on children less than two years of age to combat the early stages of severe malnutrition.

## 6. Conclusions

There is dearth of qualitative and mixed-method studies on the causes of child malnutrition in Pakistan. In terms of geographical area covered, most of the studies were conducted in the provinces of Punjab, Sindh, and Khyber Pakhtunkhwa, whereas Baluchistan, Azad Kashmir, Gilgit Baltistan, and Federally Administrated Tribal Areas (FATA) were largely neglected by researchers. The prevalence of all types of malnutrition in Pakistan was found to be higher than the global threshold value. It was found that malnutrition starts at an early age and remains persistent at later stages. Most of the researchers employed the anthropometry technique i.e., wasting (weight-for-height), stunting (height-forage), and underweight (weight-for-age) by the NCHS/WHO for assessment of child nutritional status. The most vulnerable age group of children (6–23 months) was entirely neglected to exclusively study infant and young child feeding practices. It was clearly evident that higher intensity of child malnutrition prevails in the rural areas of Pakistan. Early marriages, large family size, high fertility rates with a lack of birth spacing, low income, and the lack of breastfeeding and exclusive breastfeeding were found to be major determinants of child malnutrition. 

## 7. Strength and Limitation of Study

This is the first study to review the range of methodologies and identify the neglected geographical areas and widely used methods of previously published papers assessing child malnutrition in Pakistan. This research will be helpful for further researchers to design their methodology and target population before conducting their research. This study highlighted methodological gaps: preferred techniques for assessing the child malnutrition, varied study designs, and tagged the geographical areas with human development index rating. However, there are some limitations in this study because the previously published papers were published in different time spans, varied types of populations were under-studied, the varied age groups of the children, the small sample sizes, and different natures of the studies. One-fourth of the studies reviewed in this research had sample sizes of less than 200, and it is very difficult to generalize the result of these small-scale studies. The studies reviewed in this research had the three types of the population groups i.e., community-based, hospital-based, and school-based studies. There was much variation in results of all three types of population, and it was difficult to draw conclusion about the causes of child malnutrition. Therefore, it is suggested that the future studies must be clear in the methodological stage for the selection of the homogeneous studies in term of target population, type and nature of the study, and sample size for better understanding the problem under investigation. There is a need for more research with a more homogeneous approach to know the current real situation of child malnutrition in a socio-cultural and demographic context.

## 8. Recommendation for Future Studies

It is suggested that further studies should focus on the causes of malnutrition in the infancy stage to strengthen the nutritional status of children and that the neglected areas should also be studied to highlight this problem. The major causes of child malnutrition are also associated with the mother’s reproductive health care behavior, maternal diet, and autonomy. It is therefore suggested that further research also study the maternal factor while exploring the causes of child malnutrition. There is also a need to know about the diet and type of food that mothers prefer to give their children while exploring the causes of child malnutrition. Therefore, it is also suggested that the dietary diversity (DD) recall method should also be utilized to know about the preferred food that mothers give to their children. The component of micronutrients deficiency was entirely omitted under discussion of the previously published research. It is recommended that the micronutrient assessment should be a component of future research for a better understanding of the type of malnutrition. There is also the dire need of qualitative and mixed-method research to understand and highlight the cultural and lifestyle factors that cause child malnutrition in Pakistan.

## Figures and Tables

**Table 1:** Description of the studies included in the review.

Study	Type	Participant	Sample Size	Target Population	Malnutrition Assessment	Study Design	Rural/Urban	Human Development *	Geographical Area: Province (Village/Town/City)
Achakzai and Khan [32] (2016)	Community based	Children and mothers	104	Under five years	NCHS/WHO Z score	Cross-sectional	Rural	LHD	Baluchistan, Quetta
Hamad et al. [47] (2016)	Hospital based	Pregnant mothers	200	Not mention	NCHS/WHO Z score	Cross-sectional	Not mention	Not applicable	Pakistan, four provinces
Khan et al. [44] (2016)	Community based	Mothers and children	3964	Less than five years	NCHS/WHO Z score	Cross-sectional	Rural	LHD	Sindh, Thatta
Mahmood et al. [49] (2016)	Hospital based	Guardian	100	Under five years	NCHS/WHO Z score	Cross-sectional	Urban	MHD	Punjab, Rawalpindi
SMART, [50] (2016)	Community based	Mother-child	415	6–59 Months	MUAC	Cross-sectional	Rural	LHD	Sindh, Umerkot
Zanib and Qadir [51] (2016)	Community based	Children	385	10–14 years	BMI	Cross-sectional	Rural	MHD	Sindh, Karachi
Ali et al. [30] (2015)	Community based	Children and Parents	446	Child age 6–59 months	NCHS/WHO Z score	Cross-sectional Structured Questionnaire	Rural	LHD	KPK, Nowshera, Jalozai Camp
Khan et al. [52] (2015)	Schools	Children	684	5–14 Years	NCHS/WHO Z score	Cross-sectional	Rural	LHD	Punjab, Bahwalpur
Laghari et al. [37] (2015)	Community based	Children	511	6–59 Months	MUAC	Cross-sectional	Rural	LHD	Sindh, Sanghar
Afridi et al. [31] (2014)	Community based	Children	550	2–5 years	NCHS/WHO Z score	Cross-sectional	Rural	LHD	KPK, Swat, Mangora
Anonymous [39] (2014)	Community bases	Mother-Child	50,247	Under five	MUAC	Cross-sectional	Rural	VLHD	Sindh, Tharparkar
Ullah et al. [53] (2014)	Hospital based	Children	186	Under five children	Gomez’s Classification	Cross-sectional	Rural	LHD	KPK, Swat
Gul and Kibria [38] (2013)	Community based	Children and mothers	200	Less than three years	Weight for age using Harvard standard	Cross-sectional	Rural	MHD	KPK, Peshwar—two rural communities
Nisar et al. [35] (2013)	Hospital based	Children	116	6 months to 12 years	NCHS/WHO Z score and Hemoglobin level	Cross-sectional	Urban	VHHD	Islamabad
Rehman et al. [54] (2013)	Primary Schools	Children	400	4–12 years	BMI	Cross sectional	Urban	MHD	KPK, Peshawar
Batool et al. [34] (2012)	Primary schools	Children	432	4–12 years	Jelliffe	Cross-sectional	Rural	MHD	Punjab, Faisalabad
Mushtaq et al. [55] (2012)	Schools	Children	1860	5–12	NCHS/WHO Z score	Cross-sectional	Urban	HHD	Punjab, Lahore
Mushtaq et al. [41] (2011)	Primary schools	Children	1860	5–12 years	BMI	Cross-sectional	Urban	MHD	Punjab, Lahore
Hasnain et al. [56] (2010)	Community based	Mother and child	800	Less than five years	NCHS/WHO Z score	Cross-sectional	Rural	LHD	Sindh, Dadu
Khattak and Ali [43] (2010)	Community based	Children	140	Child age 2–5 years	NCHS/WHO Z score	Cross-sectional Structured Interview	Rural	VLHD	KPK, Swabi
Riaz et al. [57] (2010)	Primary schools	Children	344	5–10 years	BMI	Cross-sectional comparative study	Urban	MHD	Punjab, Rawalpindi
Nuruddin et al. [40] (2008)	Secondary data	Children and mothers	1533	Less than 35 months	NCHS/WHO Z score	Secondary data analysis	Rural	LHD	Thatta, Sindh
Ansari et al. [33] (2006)	Community based	Mothers	420	6–18 Months	NCHS/WHO Z score	Cross-sectional	Urban	MHD	Sindh, Karachi
Anwar et al. [58] (2006)	Primary schools	Children	1185	5–14 years	Weight and height using Jelliffe’s classification	Cross-sectional	Punjab, Sargodha	MHD	Punjab, Rural 5 villages
Khuwaja et al. [45] (2005)	Primary schools	Children	1915	6–12 years	NCHS/WHO Z score	Cross-sectional	Rural	LHD	Sindh, Rural 4 Villages
Anwar and Awan [42] (2003)	Schools	Children	2042	6–12 Years	NCHS/WHO Z score	Cross-sectional	Urban	MHD	Punjab, Faisalabad
Shah et al. [36] (2003)	Community based	Children and mothers	1878	Less than three years	NCHS/WHO Z score	Cross-sectional	Rural	LHD	Four rural districts, Sindh
Mian et al. [59] (2002)	Community based	Mothers and caretakers	200	5–10 years	NCHS/WHO Z score	Cross-sectional Quantitative and Qualitative	Urban	VHHD	Islamabad

MUAC, mid upper arm circumference; NCHS, National Center for Health Sciences; WHO, World Health Organization; Z score, standard score; KPK, Khyber Pakhtunkhwa. *VHHD (very high human development), HHD (high human development), MHD (medium human development), LHD (low human development), VLHD (very low human development).

**Table 2:** Major findings of studies included in the review.

Study and Years	Associated Factors with Child Malnutrion
Achakzai and Khan [32] (2016)	Stunting and wasting in children were 48% and 10% respectively. Socio-demographic characteristics, maternal health, and child health indicators were significantly associated with stunting and wasting.
Hamad et al. [47] (2016)	There was a greater ratio of undernutrition in boys as compared to girls.
Khan et al. [44] (2016)	The prevalence of underweight, stunting, and wasting was 39%, 48%, and 16% respectively. Boys were found to be more stunned compare to girls. Children in the poorest households were two times more like to be stunted and wasted compared to wealthier households. Diarrhoea was associated with underweight.
Mahmood et al. [49] (2016)	32% of children were malnourished. Study indicated malnutrition to be significantly associated with maternal illiteracy and presence of disabled family members in home.
SMART, [50] (2016)	30% of children belonging to rural areas were malnourished compared to 19% in urban areas. Half of the children were stunned. Children belong to 6–17 month’s age were more stunned and wasted as compare to higher age group.
Zanib and Qadir [51] (2016)	Physical abuse among the domestic child labour was the major factor of malnutrition.
Ali et al. [30] (2015)	9% of children were stunted, 11% were underweight, and 4% were wasted. Illiteracy, large family size, late and early weaning, lack of exclusive breast feeding, and poverty were the factors associated with malnutrition.
Khan et al. [52] (2015)	The childrenin nuclearfamilies have higher risk to be wasted and stunned. Mother’s education was found strong predicator of reducing the malnutrition burden in children.
Laghari et al. [37] (2015)	66% children were affected by malnutrition. Severe malnutrition was significantly higher in female children. Malnutrition was significantly higher in youngerchildren 6–23 months than in older children 24–59 months.
Afridi et al. [31] (2014)	14% were underweight; 8% of the children were wasted; while 8% were stunted.
Anonymous [39] (2014)	Girls were found to be more malnourishedcompared to boys and children in the younger age group were also severely malnourished as compare to older age group.
Ullah et al. [53] (2014)	38% male and 32% female children were malnourished. Risk factors for child malnutrition were lack of education, lack of immunization, teenage pregnancy, and large family size.
Gul and Kibria [38] (2013)	61% males and 40% females were found malnourished. 71% children less than two years were malnourished. Large family size, poor socioeconomic status, mother’s illiteracy, younger mothers, maternal anemia and multipleparities were the major causes of child malnutrition.
Nisar et al. [35] (2013)	Children admitted with nutritional anemia in children hospital, Islamabad were belonged to the less than two years of age and had very poor nutritional status.
Rehman et al. [54] (2013)	30% children were undernourished. It was found that 18% children were slightly, 10% were moderately and 2% were severely underweight.
Batool et al. [34] (2012)	Stunting and underweightwas more common in boys as compared to the girls. Low socioeconomic status, large family size, low literacy ratewas associated with poor health and nutrition in children.
Mushtaq et al. [55] (2012)	Poverty, lower education, low income, and overcrowded houses were the associated factors of children malnutrition.
Mushtaq et al. [41] (2011)	8% children were stunted and 10% children were wasted. Wasting and stunting were not significantly associated with gender.
Hasnain et al. [56] (2010)	61% children were stunned. Stunting was associated with ethnicity and birth interval less than two years.
Khattak and Ali [43] (2010)	50% of pre-school children were facing malnutrition. Strong association of child malnutriton was found with family size, household income, and number of children in family
Riaz et al. [57] (2010)	24% and 11% children were found to be underweight and stunted respectively. Stunted growth was found in 13% males and 8% female children.
Nuruddin et al. [40] (2008)	Prevalence of wasting, stunting, and underweight were higher in less than two year of children.
Ansari et al. [33] (2006)	Female children were three times more likely to be stunted than male. Food insecurity, lack of child feeding knowledge, and child health care practices were the major causes of child malnutrition.
Anwar et al. [58] (2006)	46% of school attending childrenwas malnourished. One fourth children were facing dental caries scabies and multiple boils are the common diseases of malnourished children.
Khuwaja et al. [45] (2005)	16% children were stunted. Female children compared to males were more likely to be stunted. Fathers who were working as public servant, farmers and shopkeepers were more likely to have children who were stunted when compared to landlords.
Anwar and Awan [42] (2003)	36% children were stunned and 45% were underweight. Female childrenin rural areas were found two times more malnourished as compare to females living in urban areas.
Shah et al. [36] (2003)	26% children were wasted and 15% were stunted. Mother’s illiteracy, poverty, and overcrowded houses were more likely to have stunned children.
Mian et al. [59] (2002)	Overall 44% children were malnourished. Childreninhigher age group, large family size and poverty were the majoer factors of child malnutrition.
